# Single-neuron activity and eye movements during human REM sleep and awake vision

**DOI:** 10.1038/ncomms8884

**Published:** 2015-08-11

**Authors:** Thomas Andrillon, Yuval Nir, Chiara Cirelli, Giulio Tononi, Itzhak Fried

**Affiliations:** 1 Laboratoire de Sciences Cognitives et Psycholinguistique (UMR8554), EHESS/CNRS/ENS-DEC, 75005 Paris, France; 2 Ecole Doctorale Cerveau Cognition Comportement, ENS/EHESS/ParisVI/ParisV, 75005 Paris, France; 3 Department of Psychiatry, University of Wisconsin-Madison, 6001 Research Park Blvd, Madison, Wisconsin 53719, USA; 4 Department of Physiology and Pharmacology, Sackler School of Medicine, and Sagol School of Neuroscience, Tel Aviv University, Tel Aviv 69978, Israel; 5 Department of Neurosurgery, David Geffen School of Medicine and Semel Institute For Neuroscience and Human Behavior, UCLA, 710 Westwood Plaza, Los Angeles, California 90095, USA; 6 Functional Neurosurgery Unit, Tel Aviv Medical Center and Sackler School of Medicine, Tel Aviv University, Tel Aviv 69978, Israel

## Abstract

Are rapid eye movements (REMs) in sleep associated with visual-like activity, as during wakefulness? Here we examine single-unit activities (*n*=2,057) and intracranial electroencephalography across the human medial temporal lobe (MTL) and neocortex during sleep and wakefulness, and during visual stimulation with fixation. During sleep and wakefulness, REM onsets are associated with distinct intracranial potentials, reminiscent of ponto-geniculate-occipital waves. Individual neurons, especially in the MTL, exhibit reduced firing rates before REMs as well as transient increases in firing rate immediately after, similar to activity patterns observed upon image presentation during fixation without eye movements. Moreover, the selectivity of individual units is correlated with their response latency, such that units activated after a small number of images or REMs exhibit delayed increases in firing rates. Finally, the phase of theta oscillations is similarly reset following REMs in sleep and wakefulness, and after controlled visual stimulation. Our results suggest that REMs during sleep rearrange discrete epochs of visual-like processing as during wakefulness.

## Abstract (web summary)

Since the discovery of rapid eye movements (REMs), a critical question endures as to whether they represent time points at which visual-like processing is updated. Here the authors demonstrate that cortical activity during sleep REMs shares many properties with that observed during saccades and vision.

## Graphical abstract

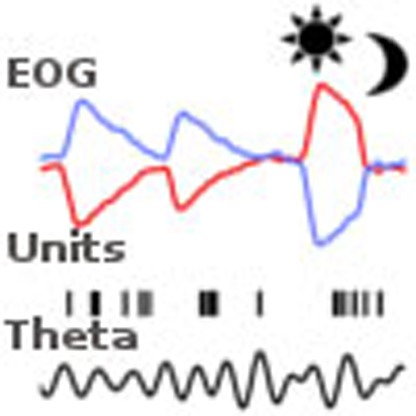

A seminal discovery in sleep research identified sleep periods with rapid eye movements (REMs), vivid dreams, electroencephalographic (EEG) activation and muscle atonia, known as REM sleep^1^,^2^,^3^. Since its discovery, a straightforward, fascinating question endures: are REMs non-specific signatures of arousability or do they represent specific times at which visual-like processing is updated?

The intuitive ‘scanning hypothesis', relating directional properties of REMs in sleep to shifts of gaze scanning the dream imagery, has proven difficult to assert. REMs have been shown to be highly similar to saccades during wakefulness in terms of their kinematic properties^4^ and we use the terms interchangeably throughout. Retrospective comparisons of REM directions with dream recall yielded highly inconsistent results^2^,^5^,^6^,^7^,^8^, reviewed in detail in ref. 9. Several lines of evidence oppose the scanning hypothesis: for example, REMs in sleep (‘s-REMs') persist in animals and humans without sight (for example, fetuses, congenitally blind who do not report visual dreams). Visual dreams also occur in Non-Rapid Eye Movement (NREM) sleep^10^ and during ∼80% of REM sleep that is devoid of REMs (‘tonic REM sleep'). Indeed, it could be that REMs reflect general arousal and are analogous to penile erections that occur throughout REM sleep, but do not necessarily imply sexual contents in dreams^11^. On the other hand, recent studies with patients who enact their dreams due to absence of muscle paralysis (REM sleep behavior disorder, RSBD^12^), suggest that, when present, REMs scan the dream scene^9^. Overall, the evidence to date is limited to relating REMs in human sleep to either reports of dream content or to overt behavior, but the underlying changes in brain activity have remained relatively unexplored.

Thus, an important and tractable question is whether s-REMs co-occur with similar modulations in neuronal activity as during wakefulness, particularly in regions associated with visual-mnemonic processing (such as occipitotemporal cortex and the medial temporal lobe (MTL)). Indeed, during wakefulness each saccade (‘w-REMs') gives rise to a discrete epoch of visual processing^13^, associated with behavioural saccadic suppression and a subsequent visual response^14^,^15^,^16^. In animals, s-REMs often coincide with stereotypical potentials termed ponto–geniculo–occipital (PGO) waves^3^,^17^,^18^,^19^. However, the relation of s-REMs with neuronal activity in high-order visual and mnemonic circuits remains unknown.

The goal of this study was to establish the relation between s-REMs and underlying activity in visual-mnemonic regions, and to compare such modulations with those occurring during waking vision. To this end, we examined the underlying intracranial EEG (*n*=172 depth electrodes) and single-unit activities around REMs during sleep and wakefulness (*n*=600) and during controlled visual stimulation (*n*=1,457) in neurosurgical epilepsy patients. The results demonstrate, for the first time, that MTL activity during s-REMs shares many properties with that observed during saccades and vision. A plausible interpretation is that s-REMs represent privileged time points at which neuronal activity and associated visual-like processing is updated.

## Results

### Rapid eye movements in sleep and wakefulness

**Figure 1:**
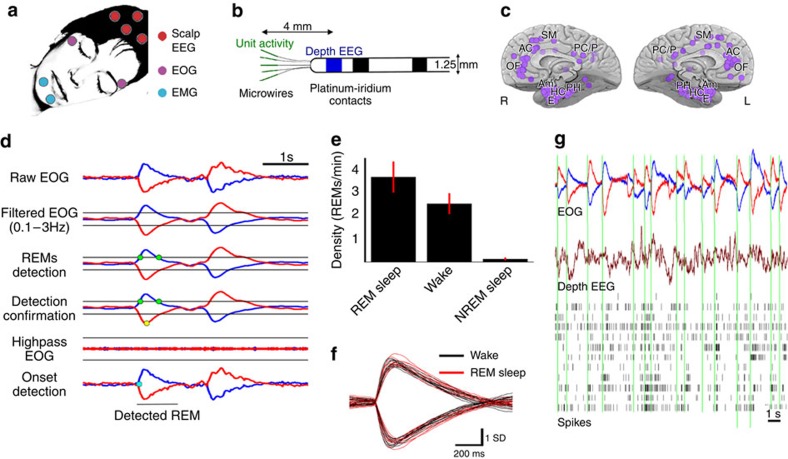
Data overview and eye movement detection. (**a**) Set-up for polysomnographic sleep and wake recordings. (**b**) Illustration of flexible probes used for recording depth EEG (blue: platinum contact) and unit activity (green: microwires). (**c**) Medial view of depth electrode locations (purple dots) spanning multiple brain regions. (**d**) REM detection: first row, raw EOG showing two typical REMs in REM sleep. Second row, band-pass filtered (0.1–3 Hz) EOG with thresholds set at mean+2 s.d. (black lines). Third row, detection of epochs crossing the threshold in one EOG channel (green dots). Fourth row, detection is confirmed with opposite polarity in second EOG channel (yellow dot). Fifth row, verification that epoch is free of epileptic spikes in high-pass filtered trace. Sixth row, visual confirmation of REM onset. (**e**) Occurrence of REMs across vigilance states (mean±s.e.m., *n*=11/12/13 in REM sleep, wake and Non-Rapid Eye Movement sleep, respectively). Note the near-absence of REMs in NREM sleep. (**f**) Average traces of REMs in each participant during wakefulness (black, *n*=12) and REM (red, *n*=11). (**g**) Example of EOGs (top, red/blue), depth EEG (brown) and unit spiking activities in the MTL (black lines) during 20 s of wakefulness. Vertical green lines depict detections of REM onsets. Note that some REMs are associated with a tendency of neurons to show transient spike-train responses shortly after REM onset. Am, amygdala; HC, hippocampus; E, entorhinal cortex; L, left hemisphere; PC/P, posterior cingulate/parietal cortex; PH, parahippocampal gyrus; SM, Supplementary motor; R, right hemisphere.

To compare neuronal activity underlying REMs in sleep and wakefulness, we conducted full-night (421±20 min (mean±s.e.m.)) polysomnographic sleep studies with adjacent epochs of wakefulness (*n*=13 participants), as well as recordings during controlled visual stimulation (*n*=9 participants) and an eye-movement paradigm (one participant), in 19 neurosurgical patients with intractable epilepsy. Participants were implanted with depth electrodes (Fig. 1a–c) that recorded depth EEG and spiking activity during sleep and wake from 600 units (355 putative single units, 245 multi-unit clusters), as described thoroughly elsewhere^20^. Stability of unit recordings across several hours was assessed in detail (Supplementary Fig. 1). Recordings also included synchronized scalp EEG, electrooculogram (EOG), electromyogram and continuous video monitoring. Sleep–wake stages were scored following established guidelines^21^. Sleep architecture and power spectra of scalp EEG were in general agreement with typical findings in healthy young adults.

REMs were detected in EOG traces in a semi-automatic fashion (see Methods section, Fig. 1d–f). REMs were selected for further analysis when at least 20 events were detected for a given subject and sleep stage (*n*=12 and 11 individuals for wakefulness and REM sleep, respectively). On an average, 3.7±0.8 and 2.6±0.5 REMs per minute were detected in REM sleep and wakefulness, respectively, while detection in NREM sleep was not significantly different from zero (Fig. 1e; *P*=0.17, Mann–Whitney *U*-test, *n*=13 patients), attesting to a low rate of false detections. The relatively low density of REMs in wakefulness likely reflects the experimental setup (overnight recordings in darkness) and EOG measurements that best capture saccades with a large horizontal component (see Supplementary Note for further details).

The morphology of REMs in sleep and wakefulness was highly similar (Fig. 1f), in line with previous studies^4^,^22^. For example, when subtracting the EOG traces of w-REMs and s-REMs, no statistical differences were found (paired *t*-test, *n*=11 pairs, *α*=0.05, False Discovery Rate (FDR) correction for multiple comparisons).

### Modulation of neuronal activity during REMs

We set out to examine if neuronal activity, especially that occurring in MTL regions, including the amygdala, hippocampus, entorhinal cortex and parahippocampal gyrus (Table 1), is robustly modulated in concert with REMs, and to what extent such activity is reminiscent of visually evoked activity in wakefulness. To this end, we compared neuronal activity during (a) REM sleep (‘s-REMs'), (b) spontaneous REMs in wakefulness (‘w-REMs') and (c) during a controlled visual stimulation paradigm in which subjects maintained fixation during short (200 ms) presentation of images. Figure 1g depicts representative traces of eye movements along with simultaneously recorded neuronal activity and depth EEG in the MTL. As can be seen, in some cases, REMs were associated with modulations in spiking activity.

**Figure 2:**
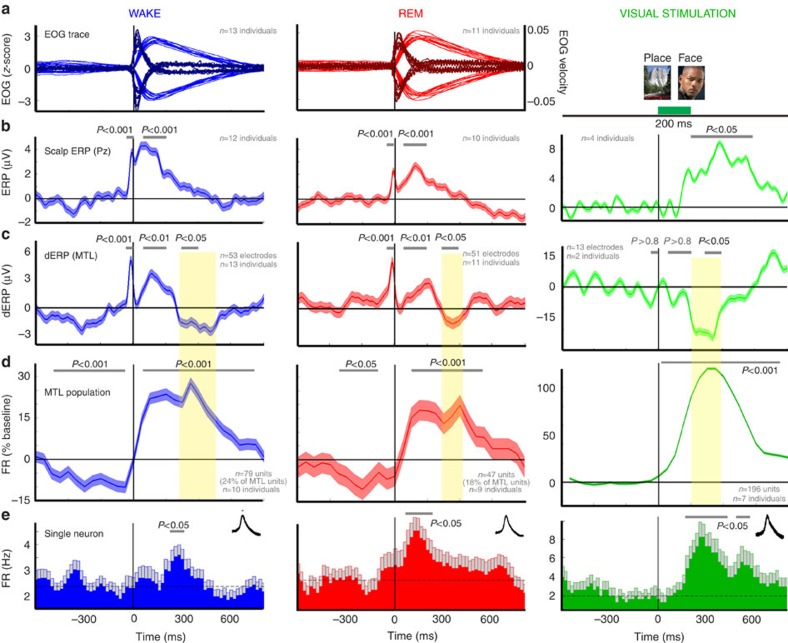
REM-triggered averaging of neuronal activity. REM-triggered averaging of multiple signals of interest in wakefulness (left) and REM sleep (middle), as well as during controlled visual stimulation (right). Intracranial recordings (**c–e**) focused on MTL regions. (**a**) Mean (± s.e.m.) waveform of EOG traces (light colours: amplitude; dark colours, velocity). (**b**) Mean scalp ERPs computed at Pz. (**c**) Mean depth EEG potentials (dERP), (**d**) mean peri-REM time histogram for ‘biphasic' MTL neurons in wakefulness (*n*=79 units, *n*=16,738 REMs), REM sleep (*n*=47 units, *n*=4,510 REMs), and visual stimulation (*n*=196 units, *n*=23,248 trials). Note the reduction in firing rate (−400 to 0 ms) and increased activity (150–550 ms) relative to REM onsets, and similar increased activity upon visual stimulation (right). Yellow shading highlights a time window in which negative components in the dERPs are associated with increase neuronal activity in all three conditions. Bars illustrate statistical deviances from baseline corrected for multiple comparisons (Methods). (**e**) Representative examples of activity in individual neurons (black inset: spike waveform) around REM onsets in wake and sleep (same neuron from parahippocampal gyrus) and visual stimulation (different neuron but from same brain region). Peri-REM-time histograms were computed on 120 ms bins (100 ms overlap). Mean firing rate of each neuron is marked with a dashed horizontal line, grey bars above illustrate statistically significant deviations from baseline (*t*-test, *P*<0.05). Error bars denote the s.e.m. across patients (**a**,**b**), dEEG channels (**c**) or REMs (**d**,**e**).

We proceeded to quantify the REM-related changes in neuronal activity across the entire data set, and further compared such activities to visually evoked responses to pictures of faces and places (*n*=133 responsive neurons out of 1,457 overall; 27 sessions in eight individuals, see Methods section). A triggered-averaging analysis was performed around REM onsets, as well as following controlled visual stimulation (Fig. 2). We examined simultaneously recorded scalp EEG (*n*=12, 10 and 4 electrodes located at Pz in wake, sleep and visual experiment, respectively), depth EEG (*n*=53 (13 patients), 51 (11 patients) and 13 (2 patients) recording sites in wake, sleep and visual experiment, respectively) and spiking activity within the MTL (*n*=437, 349 and 1,049 in wake, sleep and visual experiment, respectively).

Scalp event-related potentials (ERPs) around REMs (Fig. 2b) during both sleep and wakefulness revealed a waveform in line with previous studies^23^, consisting of an initial transient peak before saccade onset ((−25 to 0)ms, *P*<0.001, one-tailed *t*-test compared with a (−800 to −400)ms baseline) followed by a second positive component ((50–200)ms *P*<0.001). Following controlled visual stimulation, the scalp ERP exhibited a more delayed positive component ((200–600)ms, *P*<0.05; Fig. 2b, right). In the depth EEG (‘dERP'), robust evoked potentials were found across MTL regions in relation to REMs with a similar positivity around 150 ms as found in the scalp EEG (*P*<0.01, Fig. 2c). This component was absent during visual presentation without eye movements (*P*>0.8, Fig. 2c, right). Notwithstanding, a late negativity ((300–400)ms, *P*<0.05) was observed within the MTL after REMs in sleep and wakefulness, as well as after the presentation of images with fixation. Since depth EEG signals were referenced to ear lobes (thereby attenuating ocular artifacts^24^), the presence of robust dERPs in the MTL (and especially components occurring hundreds of milliseconds after eye movement onsets) suggests an underlying modulation of neuronal activity upon REMs in both sleep and wakefulness, whose late component is reminiscent of visual-evoked activity.

Most importantly, single-unit spiking activity was robustly modulated around REMs, and this modulation was highly similar across wakefulness and REM sleep. A pattern consisting of a reduction in firing rate just before REM onsets, followed by subsequent increased spiking was observed in the average activity of all recorded units in wakefulness. A similar albeit weaker modulation was likewise observed in the average activity across all units during REM-sleep (Supplementary Fig. 2a, *n*=412 and *n*=318 in wakefulness and sleep, respectively). We searched for neurons whose activity was significantly modulated in any direction and at any time interval around REMs (see Methods section and Supplementary Fig. 2 for full details of selection process). From this unbiased approach, the stereotypical bi-phasic pattern of activity emerged in both sleep and wakefulness (Supplementary Fig. 2b). We then focused on the units showing this bi-phasic pattern in MTL regions specifically for further analysis (see Methods section and Fig. 2d,e for MTL population average and representative neurons, respectively) since neurons that showed bi-phasic modulations around REMs were more readily observed in the MTL (Table 1, 24% of all MTL units in wake (*n*=79 in 10 patients) and 18% in REM sleep (*n*=47 in 9 patients)) compared with frontal lobe regions (13% in wake and 12% in REM sleep). In contrast, non bi-phasic modulation profiles were observed as frequently across regions (Table 1). Along the same line, when searching for bi-phasic activity profiles around random time points (instead of real REMs), a significantly lower proportion of MTL neurons was found (one-tailed paired Mann–Whitney *U*-test, *P*<0.01, see Supplementary Material) but the proportion of frontal lobe neurons did not show such a difference (*P*=0.9). The higher proportion of bi-phasic modulations in the MTL argues against a global non-specific activity pattern.

Finally, the temporal dynamics of increased MTL firing following REMs was reminiscent of neuronal responses in the controlled visual experiment (Fig. 2d,e, right), although these were of stronger amplitude (*P*<0.001)—a difference to be expected given that stimuli were specifically selected to drive strong responses in the recorded neurons (Methods section). On an average, around REMs, bi-phasic neurons reduced their firing by 11% in sleep and 7% in wake at −200 ms (*P*<0.005, *t*-test) and increased their firing by 17% in sleep and 22% in wake at +250 ms (*P*<0.001, *t*-test). The post-REM increase coincided in time with the negativity observed in the dERPs. The pre-REM reduction in firing was unrelated to effects of previous REMs, as it was similarly observed also when focusing on ‘isolated' REMs that did not occur in succession (not shown). In addition, visual responses were substantially later in time, peaking at 300 ms after image onset, whereas post-REM activity peaked around 250 ms after REM onset (equivalent roughly to 150 ms after eventual fixation^25^). In summary, REMs in both sleep and wakefulness were associated with multiple signatures of transient neuronal activities in the MTL that were similar across vigilance states and reminiscent of visually evoked activity.

**Figure 3:**
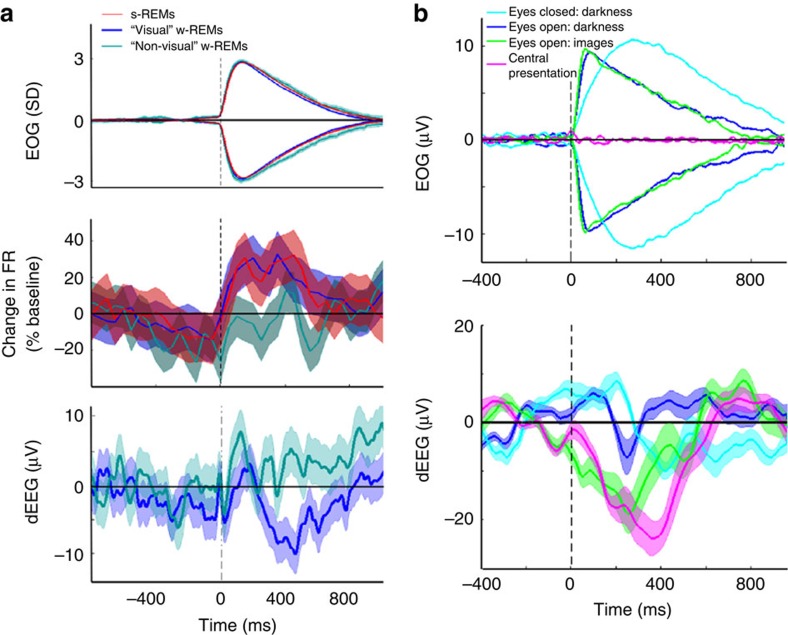
Comparison of neuronal activity underlying visual and non-visual REMs. (**a**) REM-triggered averaging of single-unit firing rate in wakefulness, separately for epochs tagged as ‘non-visual' (open eyes in a dark room, green) or ‘visual' (patient watching TV/DVD or interacting with people in a well-lit room, blue). s-REMs during REM sleep are superimposed for comparison (red). Top: mean waveform of EOG traces for ‘visual' w-REMs (blue), ‘non-visual' w-REMs (green), and s-REMs (red) could not reveal differences between conditions. Middle: average peri-REM-time-histogram (‘visual': *N*=79 units and 1,250 REMs; ‘non-visual': *N*=47 units and 140 REMs) expressed in percentage change of baseline. Statistical analysis matched for sample size (*n*=140 REMs in both conditions) revealed a significant decrease in firing rate prior to REM onsets for both ‘visual' and ‘non-visual' epochs, whereas a significant increase was observed following REM onsets only for ‘visual' w-REMs (‘visual': *P*<0.05, ‘non-visual': *P*=0.33, Mann–Whitney *U*-test). Bottom: dERP time-locked to REMs onset (‘visual': *N*=287 REMs in 70 channels, ‘non-visual': *N*=287 REMs in 50 channels). Number of REMs were equated across conditions (*n*=287). Note the negative deflection between 200 and 500 ms for ‘visual' REMs only. Error bars show s.d. across REMs. (**b**) Average EOG and intracranial dERPs in one participant when performing saccades in darkness with eyes closed (cyan, *N*=42), eyes open in darkness (dark blue, *N*=47), when performing saccades to pictures presented at the periphery (green, *N*=55) or when being presented with images directly at the center of fixation (magenta, *N*=59). Top: EOG traces corresponding to the four different experimental conditions. Note the major difference in shape between the eyes closed and eyes open conditions. Bottom: MTL dERPs time-locked to the onset of saccades/image presentation. Error bars show one s.d. Note that while evoked responses following image presentation and saccades appear similar, saccades in darkness reveal a much weaker or absent modulation.

To further investigate to what extent REM-related effects were driven by motor aspects of eye movements (‘corollary discharge') versus visual-like aspects, we further divided wakefulness into putatively ‘visual' and ‘non-visual' periods based on behavioural notes (for example, watching television or meeting family in a well-lit room as ‘visual' versus lying with open eyes in a dark room as ‘non-visual', see Supplementary Methods). The rate of w-REMs in ‘visual' periods was significantly greater than in ‘non-visual' periods (14.7±14.6 versus 0.7±2.8 REMs per minute, *P*<0.05, Mann–Whitney *U*-test). Analysis of evoked potentials (Fig. 3) revealed that both ‘visual' and ‘non-visual' epochs were associated with a significant suppression of firing rates prior to REM onsets (difference from baseline: *P*<0.01, between ‘visual' and ‘non-visual': *P*=0.09, Mann–Whitney *U*-test, *N*=110 units and 140 REMs across eight patients). However, only ‘visual' epochs showed a robust increase in neuronal activity after REM onsets within the MTL (difference between ‘visual' and baseline: *P*<0.05; ‘non-visual' and baseline: *P*=0.33). Similarly, dERPs of ‘visual' and ‘non-visual' differed in their late negative potential (Fig. 3a), which was present only for ‘visual' REMs (significant difference observed 375–475 ms after REM onset, *t*-test, *P*<0.05, FDR correction). Moreover, a controlled paradigm during wakefulness in one individual further compared neuronal modulations occurring around w-REMs with closed eyes or during darkness with saccades towards visual targets (or passive fixation). Interestingly, already at the level of EOG waveforms, eye movements with open eyes and those occurring during REM sleep were highly similar, whereas w-REMs with closed eyes during wakefulness had markedly slower kinematics (Fig. 3b). Intracranial-evoked potentials in the MTL revealed a negative deflection following image presentation that also resembled those found after REMs (shown in Fig. 2c). This negative deflection was significantly attenuated during w-REMs without visual input (Fig. 3b). Taken together, these results suggest that neuronal modulations within the MTL after REMs cannot be solely explained by a motor corollary discharge (see also Discussion).

### Spike-train latency, selectivity and theta phase reset

Given that the dominant pattern of neuronal activity around s-REMs included a transient increase in firing rate in the few hundred milliseconds following REM onsets, it was of interest to determine to what extent the detailed features of this activity resembled visual processing in wakefulness. In particular, a robust and recently described feature of human MTL responses to visual stimulation is the significant correlation between the latency and the selectivity of neuronal spike trains, reflecting a hierarchical processing mode: a longer latency from image presentation is more readily found in neurons responding to fewer images^26^. We thus examined the presence and precise onset of spike trains in individual trials following spontaneous REMs in sleep and wakefulness, as well as following controlled visual stimulation.

**Figure 4:**
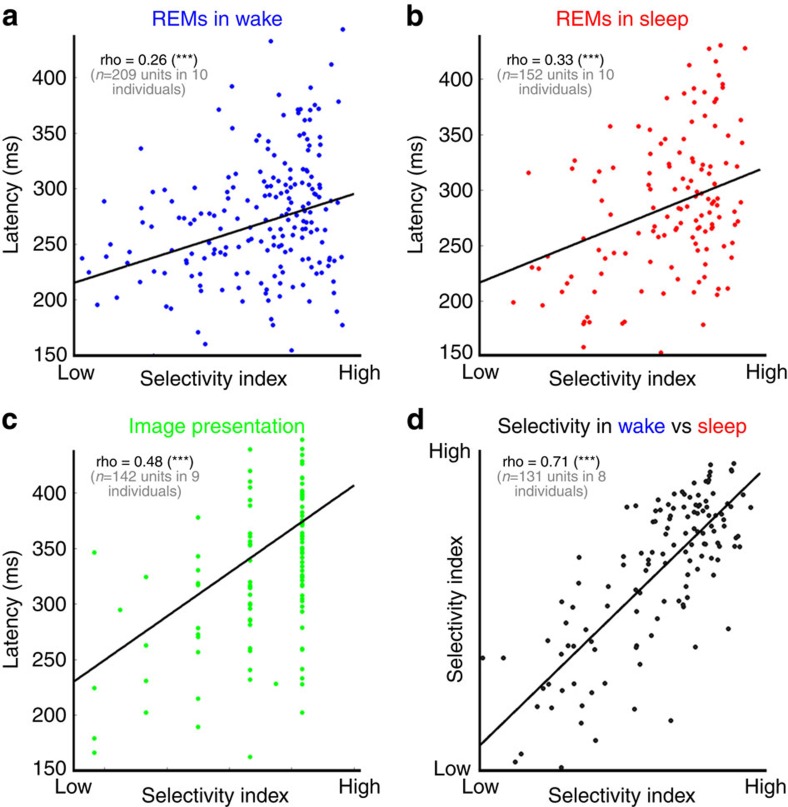
Comparison of transient spike-train properties in REM sleep and wakefulness. (**a**–**c**) Scatter plot of spike-train latency (ordinate) versus selectivity (abscissa, percent of trials eliciting a spike-train after REMs or image presentations) in w-REMs (**a**), s-REMs (**b**) and in the visual stimulation experiment (**c**). Low selectivity: neurons responds to all REMs/pictures; high selectivity: neurons responds to few REMs/pictures. Selectivity in **c** is restricted to a limited number of discrete values due to the use of six images in the image presentation experiment. Note that in all three conditions a robust correlation was observed, indicating the existence of hierarchical processing along the visual-mnemonic axis. Such correlation could not be revealed in random time points during REM sleep and wake. (**d**) Selectivity following REMs is correlated in the same neurons across wakefulness and sleep (Spearman's rank correlation, *r*=0.71, *P*=10^−23^; partial correlation taking average firing rate into account: *r*=0.51, *P*=10^−11^). ***P<0.001.

In MTL neurons, spike trains were detected in 23.3±1% (mean±s.e.m.) and 19.6±1% of REMs in wakefulness and sleep, respectively (*N*=209 and 152 units, respectively). Despite the small subset of REMs associated with spike trains, these events accounted well for the overall increase in firing rate (see Supplementary Fig. 3). Moreover, the increase in firing rate for REMs associated with spike trains was correlated across vigilance states even when regressing out the mean firing rate of each unit (Spearman's partial correlation: *r*=0.28, *P*<0.01, *n*=131 units in eight patients). Furthermore, we found a significant correlation between the average latency of firing rate increase in each MTL neuron (after REM onset) and its selectivity (tendency to increase firing rates only after a small subset of REMs, Methods section). Figure 4a–c present this correlation for w-REMs (Spearman's rank correlation, *r*=0.26, *P*=10^−4^, *n*=209 in 10 patients), s-REMs (*r*=0.33, *P*=10^−6^, *n*=152 in 10 patients), and controlled visual stimulation (*r*=0.48, *P*=10^−8^, *n*=142 in 9 patients). Crucially, when applying the same analysis on random time points during REM sleep (not associated with eye movements), latency and selectivity were not significantly correlated (*P*=0.3 and *P*=0.9 for sleep and wake), indicating that this relation occurred at specific moments, rather than reflecting basic properties of neuronal firing such as the average discharge rate. Moreover, in those cases where the same neurons were recorded in both wakefulness and REM sleep, the selectivity of neurons was highly correlated across vigilance states (*r*=0.71, *P*=10^−23^, *n*=131 units in eight patients, Fig. 4d) such that neurons that were highly selective in their visual responses in wake also showed spike trains following few s-REMs. This relation was conserved even when taking into account the average firing rate of these neurons (partial correlation: *r*=0.51, *P*=10^−11^, *n*=131 units in eight patients). The average latencies of neurons were also correlated across vigilance states (*r*=0.20, *P*=0.02, *n*=131 units in eight patients); however, this correlation was not significant when regressing out the average firing rate (partial correlation: *r*=0.09, *P*=0.3, *n*=131 units in eight patients).

**Figure 5:**
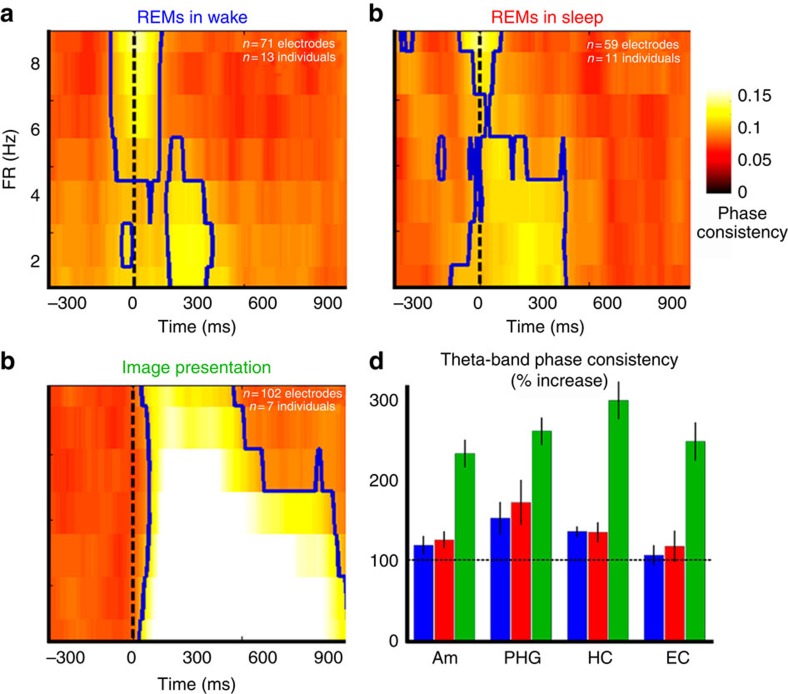
Reset of field oscillation phase following REMs and visual stimulatio*n*. (**a**–**c**) Phase consistency of intracranial dEEG within the MTL across time and frequency around w-REMs (**a**, *N*=71 channels), s-REMs (**b**, *N*=59 channels) and in the visual stimulation experiment (**c**, *N*=102 channels). Note that a significant increase within the lower theta-band (2–6 Hz) can be seen in all conditions from 0 to ∼400 ms (blue lines: **a**,**b**: *P*<0.05; **c**: *P*<10^−4^, False Discovery Rate correction). Following controlled image presentation a stronger phase reset is observed and the maximal effect occurs at similar time-frequency windows. (**d**) Phase coherence of dEEG by MTL region within the theta-band (2–6 Hz) following REMs in wakefulness (blue) and during REM-sleep (red) compared with controlled image presentation (green). Error bars show s.e.m. across channels. Phase coherence was averaged during the first 400 ms following REMs or image onset.

Finally, it is well established that theta (4–8 Hz) oscillations prevail in the MTL during both wakefulness and REM sleep^27^ and that the phase of theta oscillations is reset with engagement of visual-mnemonic mechanisms in humans^28^ and monkeys^29^. We found comparable phase resets around 2–6 Hz (‘human theta', see ref. 30) in s-REMs and w-REMs. A brief and broadband reset was visible at REM onsets (potentially reflecting a myogenic contamination) followed by a delayed, sustained and frequency-specific increase in phase coherence around 2–6 Hz (see Methods section and Fig. 5a,b). Such theta-band coherence was correlated across vigilance states (Pearson's *r*=0.27, *P*<0.05, see Supplementary Methods). Following controlled visual stimulation, stronger phase reset was observed in the MTL and the maximal effect occurred at similar time–frequency windows (Fig. 5c). When considering MTL regions separately (Fig. 5d), hippocampus and parahippocampal gyrus showed strong phase resets, while the entorhinal cortex did not show phase coherence above chance levels. These effects were consistant across vigilance states (analysis of variance, effect of regions: *P*<0.005, F=4.8; effect of state: *P*=0.4, F=0.6). Similar differences between the theta-phase coherence across MTL regions were observed during controlled imaged presentation. The stronger and delayed effects during controlled visual fixation (in comparison with REMs) mirrored those found in dERPs and single-unit firing rates (Figs 2 and 3). Thus, the temporal dynamics of MTL activity were similar during REM sleep and during visual processing in wakefulness, possibly reflecting a partial conservation of the hierarchical processing in the visuo-mnemonic pathway.

## Discussion

Despite decades of intense interest and debate, it has remained difficult to determine to what extent eye movements in REM sleep modulate brain activity. In this study, we show for the first time that in human REM sleep s-REMs were associated with transient bi-phasic modulations of spiking activity in the MTL associated with evoked potentials in depth EEG signals. A significant subset of MTL neurons exhibited a transient spike train immediately following some s-REMs, as is the case during wakefulness. Indeed, multiple properties of this activity—including spike-train latency, selectivity and the phase of simultaneous theta oscillations—were comparable during sleep and wakefulness and reminiscent of visual-mnemonic responses.

It is likely that dERPs co-occurring with modulations of neuronal activity are closely related to PGO waves, a hallmark of REM sleep^19^,^31^,^32^. PGO waves are sharp-field potentials occurring concomitantly with s-REMs and were first observed in the pons, lateral geniculate nucleus and occipital cortex of cats. Recent studies described similar phenomena in humans^33^,^34^,^35^,^36^. Originally, PGO waves were believed to occur exclusively during sleep and only in the aforementioned brain structures^3^,^17^, but subsequent evidence suggested that they are akin to visually evoked potentials^37^,^38^ and that PGO potentials similar to the current dERPs occur across wide cortical territories^39^. Although our electrode locations cannot unequivocally confirm the relation with PGO waves, it seems likely that the evoked potentials time-locked to s-REMs are closely related to PGO potentials.

The transient reduction in MTL neuronal activity immediately before REM onsets could be related to saccadic suppression^40^, a behavioural phenomenon reflecting a transient increase in visual perception thresholds during saccades^41^,^42^. Saccadic suppression was initially attributed to degraded retinal input, but later studies pointed to a more complex process^16^. However, the decreases in the activity observed here may be somewhat earlier and longer in duration than to be expected, and future studies are needed to establish this connection.

Despite the resemblance between neuronal activity around REMs and following controlled visual presentation, post-REM modulation in both sleep and wake occurred earlier than that occurring during visual processing with fixation. In the ventral visual stream of awake macaques (V4, IT), visual responses during fixation are typically observed after 100–200 ms (ref. 43). In contrast, REMs in monkeys are associated with earlier modulations shortly after eventual fixation^44^,^45^,^46^, in line with the difference observed here. Why is activity around REMs earlier than during passive vision? One possibility is that the observed neuronal modulations reflect some form of ‘attentional priming' (see below), rather than visual processing. Alternatively, during REMs, some predictive expectation already exists and drives earlier visual activity in the neurons that will participate in representing the new image.

It is important to explicitly acknowledge several limitations of the present study. First, recordings were obtained in patients with refractory epilepsy. However, great care was taken to discard data recorded in close proximity to seizures, from epileptogenic regions, and to avoid REMs temporally close to interictal epileptic spikes (see Methods section). In addition, it is well established that paroxysmal activities are far less prevalent during REM sleep^47^. Finally, sleep architectures and grapho-elements were quantitatively and qualitatively within the range of those observed in healthy individuals^20^,^48^. Second, dream reports were not collected. Nevertheless, given that the vast majority of awakenings from REM sleep yield vivid dream recalls^10^, it seems reasonable to assume that REM episodes analyzed here were likewise associated with dreaming. It is, however, important to note that dreams also occur during NREM sleep^10^, hence REMs may not be necessary for dream imagery. In addition, we were only able to acquire data in one individual during negative-control paradigms in wakefulness (that is, REMs during eye closure or darkness), however, the available data as well as retrospective analysis based on behavioural notes yielded significant differences. Finally, electrode locations were determined exclusively by clinical criteria and therefore it was not possible to examine activity in regions where PGO waves occur, or in brain regions implicated in saccade preparation. Indeed, neuronal dynamics in motor preparation centres giving rise to REMs may differ in sleep and wakefulness^49^.

What is the functional significance of the current results? One possible explanation is that such modulation reflects a motor corollary discharge driven by oculo-motor networks, but several arguments suggest that this is an insufficient explanation: (a) the post-REM increase in spiking activity during sleep exhibits several detailed characteristics observed also during wakefulness (and upon visual processing), and therefore cannot be dismissed as a non-specific effect. Accordingly, individual neurons with longer spike-train latencies after s-REMs also exhibited greater selectivity, as in vision^26^. Both the magnitude and the selectivity of the response in individual units were stable across sleep and wakefulness. Moreover, such spike-train responses were associated with a phase reset of MTL theta oscillations; (b) we found different neuronal modulations after w-REMs associated with vision in comparison with non-visual contexts such as darkness (Fig. 3). Along this line, the late (‘lambda') component following saccades in human scalp ERPs is abolished in total darkness^50^. Although in monkeys w-REMs during darkness are also accompanied by a bi-phasic modulation of firing rates in occipitotemporal cortex and MTL, such modulation is weaker than that found during vision, as we observe here^46^,^51^,^52^,^53^,^54^,^55^,^56^,^57^; (c) the fact that effects were strongest in the MTL (where retinotopic (re)mapping plays a minor role and where activity is tightly correlated with visual-mnemonic representations in humans) seems incompatible with a motor-only interpretation; (d) the timing of human MTL activity following REMs is still later than that following REMs in homologous macaque cortex as observed during visual processing. It seems more reasonable to expect such timing differences across species for visual-like, rather than motor, phenomena.

A second possibility is that REMs transiently increase cortical excitability and represent attentional shifts that ‘reset' processing frames^58^,^59^,^60^. Brief pre-REM decreases in neuronal activity may prepare the ground for subsequent processing by increasing sensitivity to inputs and amplifying responses, thereby enhancing the signal-to-noise ratio. In addition, the observed neuronal modulations co-occurred with a theta-phase reset in the MTL, as in monkeys^61^. Modulations of hippocampal oscillatory activity in primates was found to be predictive of subsequent recognition in wakefulness^28^,^29^,^62^. In this context, our findings support the notion that theta in primate MTL may play an important role in exploration of the visual (or mental) space^63^. While the underlying mechanisms remain unclear, cholinergic modulations associated with PGO waves and theta oscillations may play an important role^64^,^65^. The fact that peri-saccadic modulations in both sleep and wakefulness are earlier than those found during vision may further support the ‘attentional reset' interpretation.

Finally, given that activity in the human MTL is tightly correlated with visual awareness^66^, the current results may imply that REMs during sleep reflect a change of the visual imagery in dreams, but future studies are needed to unequivocally establish this possibility. In summary, the results establish that eye movements in REM sleep are associated with successive ‘micro-states' of cortical activity^67^,^68^ and represent transitions between discrete epochs of visual-like processing. These findings open new paths for exploring in more detail the prospective roles of eye movements and intrinsic brain activity during sleep.

## Methods

### Subjects, data acquisition and experimental paradigms

General procedures related to data acquisition have been described in detail elsewhere^20^. Here we examined 19 neurosurgical epilepsy patients (age 19–52 years, 11 females). Patients provided written informed consent prior to the participation in the research study, under the approval of the Medical Institutional Review Board at UCLA. Thirteen patients participated in full-night sleep studies, which also included epochs of wakefulness. Sleep-wake stages were scored off-line according to established guidelines^21^. In each participant, 8–12 depth electrodes recorded intracranial depth EEG and single-unit activities, along with continuous video, EOG, electromyogram and scalp EEG from four scalp derivations, as in ref. 20. Eight participants (four participated in the sleep studies) also performed a visual object recognition paradigm (*n*=27 sessions) that included 2 blocks × 12 min, when 24 trials × 12 pictures of famous people and landmarks were presented for 200 ms on a laptop, while participants performed a categorization task. One individual participated in a paradigm during wakefulness directly comparing activity during w-REMs in darkness, with closed eyes, towards visual targets and during visual stimulation with fixation. Spike sorting was performed offline separately for spontaneous sleep/wake data (*n*=600) and for visual stimulation sessions (*n*=1,334) using ‘wave_clus'^20^,^69^.

### Eye movement detection

Eye movements were detected through EOG recordings. Although indirect, this measure can be used to reliably infer about saccadic eye movements in both open eyes and closed eyes conditions (see Supplementary Note). EOG traces were obtained using two electrodes pasted below the left and above the right canthi, referenced to contralateral ear lobe signals. REMs were detected in a semi-automatic manner (Fig. 1d) by applying a bandpass filter (0.1–3 Hz), setting a detection threshold (mean+2 s.d.) and verifying: (a) duration<1.5 s, (b) opposite polarity across the two leads (corresponding to horizontal movements), (c) maximal slope>1 μV ms^−1^ and (d) no interictal spikes within 0.5 s. These parameters were chosen after careful visual examination of detected REMs. REM onsets were defined automatically as the first crossing of the two raw EOGs traces before the crossing of the detection threshold. All REM detections were verified visually. In addition, random time points were selected from the same 10-s segments but at least 4 s away from any EOG deflections, serving as control epochs devoid of any ocular activity but matched in terms of occurrence rate and position within the ultradian cycle. Latency and selectivity were computed from these random time points as a control and then compared with real REMs onsets (see Results section).

### REM-triggered averaging

Evoked potentials were obtained by averaging scalp EEG (ERP) and depth EEG (dERP) signals around REM onsets, after preprocessing (notch filter at 60 Hz and harmonics, band-passed between 0.1 and 30 Hz), mean subtraction (using a (−800 −400)ms interval), selecting epochs devoid of interictal spikes and smoothing with a Gaussian kernel of 5 ms (80 ms in Fig. 3b). In addition, for each unit separately, an average peri-REM time histogram (PRTH) was computed using 100 ms bins (50 ms overlap, see Supplementary Methods). A mean PRTH across all neurons (Supplementary Fig. 2a), all modulated neurons (Supplementary Fig. 2b) and all bi-phasic units (Supplementary Fig. 2c) was computed after normalizing each neuron's PRTH (% of baseline activity at (−800 −400)ms). Due to the predominance of bi-phasic units within the MTL, subsequent analyses (as shown in Figs 2d and 3a) were only performed for neurons within the MTL exhibiting significant bi-phasic modulations around REMs (see also Supplementary Methods).

### Analysis of spike-train properties

Occurrence and latencies of spike-trains in individual trials (REMs or images) were determined by a Poisson spike-train analysis as in ref. 70, separately for s-REMs, w-REMs and following controlled visual stimulation. Selectivity was defined as: 100%—the percent of trials (either REMs or visual stimuli) for which a spike train was detected. Comparison between individual neurons in wake and sleep (*n*=131, Fig. 4d) focused on those neurons that were recorded in both vigilance states.

### Theta-phase consistency

Consistency of the phase in the time–frequency domain was determined by extracting the phase of EEG oscillations for each REM/image. Then, for each frequency-bin and time-interval, we estimated the consistency of the phase across REMs/images for a given dEEG channel. Statistical differences were evaluated by comparing each time and frequency point to baseline values and correcting for multiple comparisons (Supplementary Methods).

## Additional information

**How to cite this article:** Andrillon, T. *et al*. Single neuron activity and eye movements during human REM sleep and awake vision. *Nat. Commun*. 6:7884 doi: 10.1038/ncomms8884 (2015).

## Supplementary Material

Supplementary Information Supplementary Figures 1-6, Supplementary Note 1, Supplementary Methods and Supplementary References

## Figures and Tables

**Table 1:** Bi-phasic single-unit modulation around REMs is more prevalent in the MTL compared with frontal regions.

Regions	Number of recorded units		% of modulated units		% of biphasic units		% of REMs with spike-trains	
	w-REMs	s-REMs	w-REMs	s-REMs	w-REMs	s-REMs	w-REMs	s-REMs
*MTL*								
Am	61 (6)	45 (5)	34.4 (3)	17.8 (3)	34.4 (5)	20.0 (4)	23.7	19.5
PHG	97 (7)	78 (6)	25.8 (6)	30.8 (5)	23.7 (6)	20.5 (6)	20.7	19.3
HC	100 (11)	80 (9)	27.0 (10)	31.3 (9)	19.0 (9)	15.0 (4)	20.8	16.1
EC	78 (6)	59 (5)	28.2 (6)	30.8 (6)	20.5 (6)	18.6 (5)	28.7	23.2
*Total*	336	217	28.3 (**)	27.9 (**)	23.5 (**)	18.3 (**)	23.5	19.5
*Frontal*								
AC	69 (6)	32 (5)	30.4 (4)	27.4 (3)	15.9 (4)	8.1 (3)	23.5	15.7
SMA	37 (3)	37 (3)	18.9 (1)	21.6 (2)	8.1 (2)	10.8 (2)	32.0	26.7
OFC	54 (6)	47 (5)	18.5 (4)	31.9 (4)	13.0 (3)	17.0 (5)	27.4	23.5
*Total*	160	146	23.8 (*)	27.4 (*)	13.1 (ns)	11.6 (ns)	27.6	22.0

The number of recorded units by brain region and vigilance state is depicted (first column) along with the proportion of neurons modulated in any direction (second column) and those presenting a bi-phasic pattern of activity (activity decreases before REMs and transient increases after REMs, third column). For each region and each column, we indicated in parenthesis the number of patients in which these units were recorded. Statistical significance was established by comparing these proportions with those obtained for random time points (paired Mann–Whitney *U*-test between real and surrogate proportions, *n*=8 and *n*=6 pairs in the MTL and frontal lobe, respectively). Note that significantly higher proportions of bi-phasic firing patterns were found only in MTL regions (*P<0.05, **P<0.01) (NS, not significant: *P*≥0.05). Fourth column: the proportion of REMs associated with transient spike-train responses (see Supplementary Methods). On an average, such spike-train responses were detected only for a minority of REMs.
