# Supplementary material for: Single-neuron activity and eye movements during human REM sleep and awake vision
Source: Nat Commun. 2015 Aug 11;6:7884. doi: 10.1038/ncomms8884 (PMC4866865; doi:10.1038/ncomms8884)
Supplement: Supplementary Information — Supplementary Figures 1-6, Supplementary Note 1, Supplementary Methods and Supplementary References [file ncomms8884-s1.pdf]

Supplementary Figures

Supplementary Figure 1. Stability of unit recordings and identification

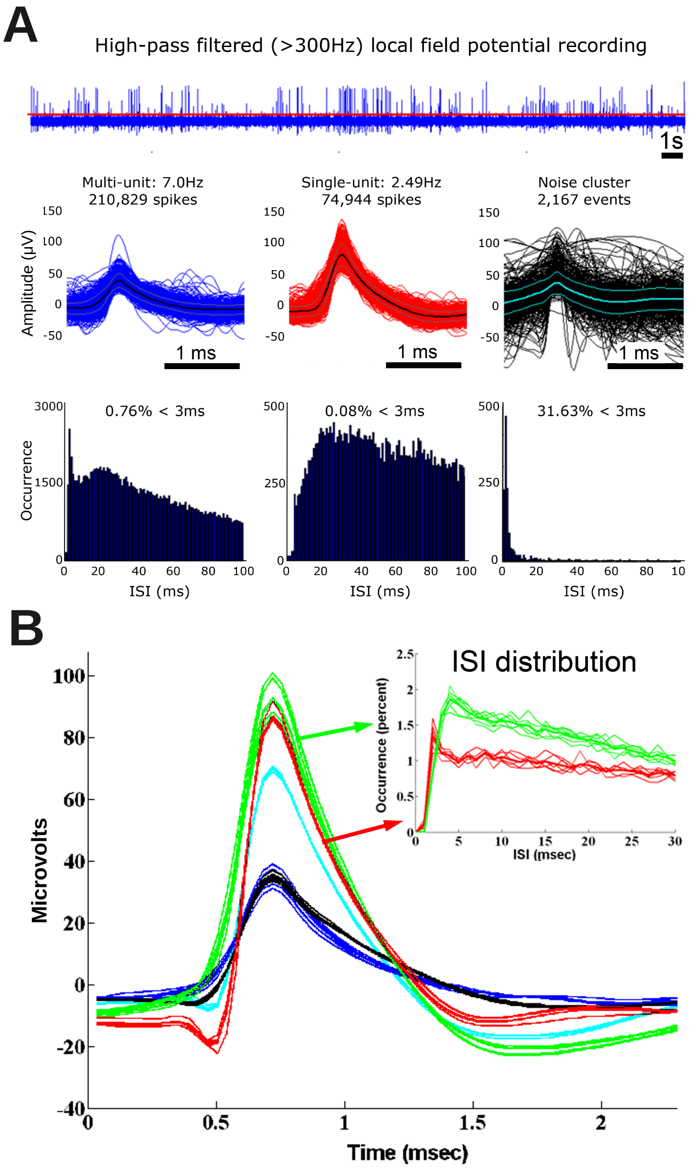

(A) Illustration of the unit identification scheme. Top row shows the high-pass filtered LFP along with the threshold for spike identification (5 SDs above the noise level (red line)). Middle row depicts action potential waveforms for three identified clusters. Bottom row shows the distribution of inter-spike-intervals (ISIs). Identification was based on the consistency of waveforms and the occurrence of ISIs within the expected refractory period (<3ms). Units were categorized either as multi-unit (blue), single-unit (red) or noise clusters (black). (B) Across long-duration recordings (~7h), waveforms and ISI distributions were consistent and separable as illustrated here by the mean action potential waveforms for 5 distinct units recorded in the same individual. Each color denotes a different unit and each trace denotes the mean waveform in separate 1h intervals throughout sleep. Inset depicts ISI distributions in separate 1h intervals for two units.

Supplementary Figure 2. Population neuronal activity shows a bi-phasic modulation around REMs in wakefulness and REM sleep.

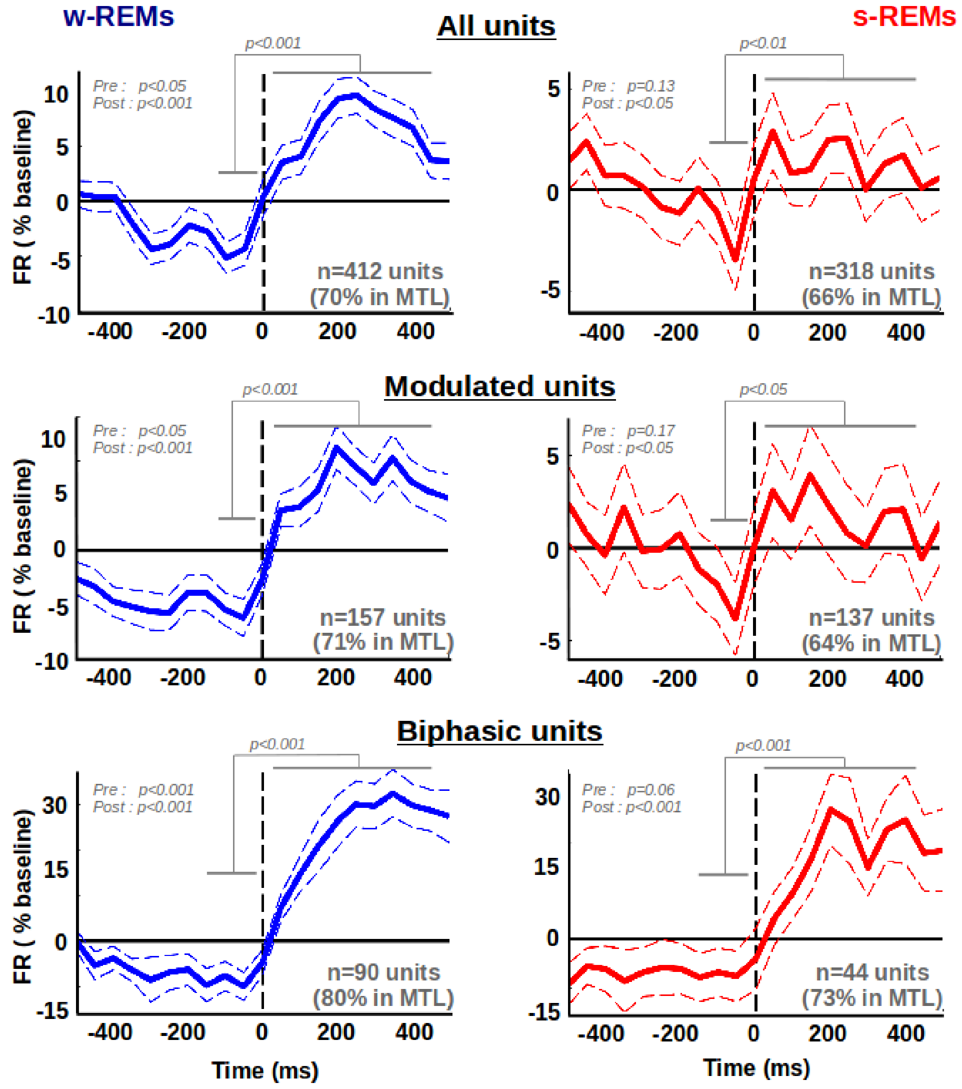

Bi-phasic neuronal responses to REM in wakefulness (w-REMs, blue, left panels) and REM-sleep (s-REMs, red, right panels) for all recorded units (A, top row), units showing significant modulations of their firing rate around REMs at any time and in any direction (B, middle row, see Methods) and units showing the specific bi-phasic pattern (C, bottom row). Units from all recorded brain regions were analyzed (see Figure 2 for an analysis focusing only on MTL neurons). Note that in both wake and sleep, a bi-phasic pattern emerges with a reduction in firing rate prior to REMs onset ([-100, -50]ms) and an increase immediately after REMs onset ([50, 400]ms). The significance of the modulation was assessed by comparing across units (one-tail paired t-tests) the activity after REM onsets to (i) the activity prior to REM onsets (see gray bars) or to (ii) baseline activity ("pre": [-100, -50]ms<baseline, "post": [50, 400]ms>baseline, see p-values reported on each panel). The number of units from all brain regions in each group (all, modulated and bi-phasic) is indicated as well as the proportion of these units located within the MTL (for an analysis focusing on MTL units, see Figure 2). Note the increase in MTL proportion for bi-phasic units. Error-bars show the SEM across units.

Supplementary Figure 3. Selective responses of units to images and REMs

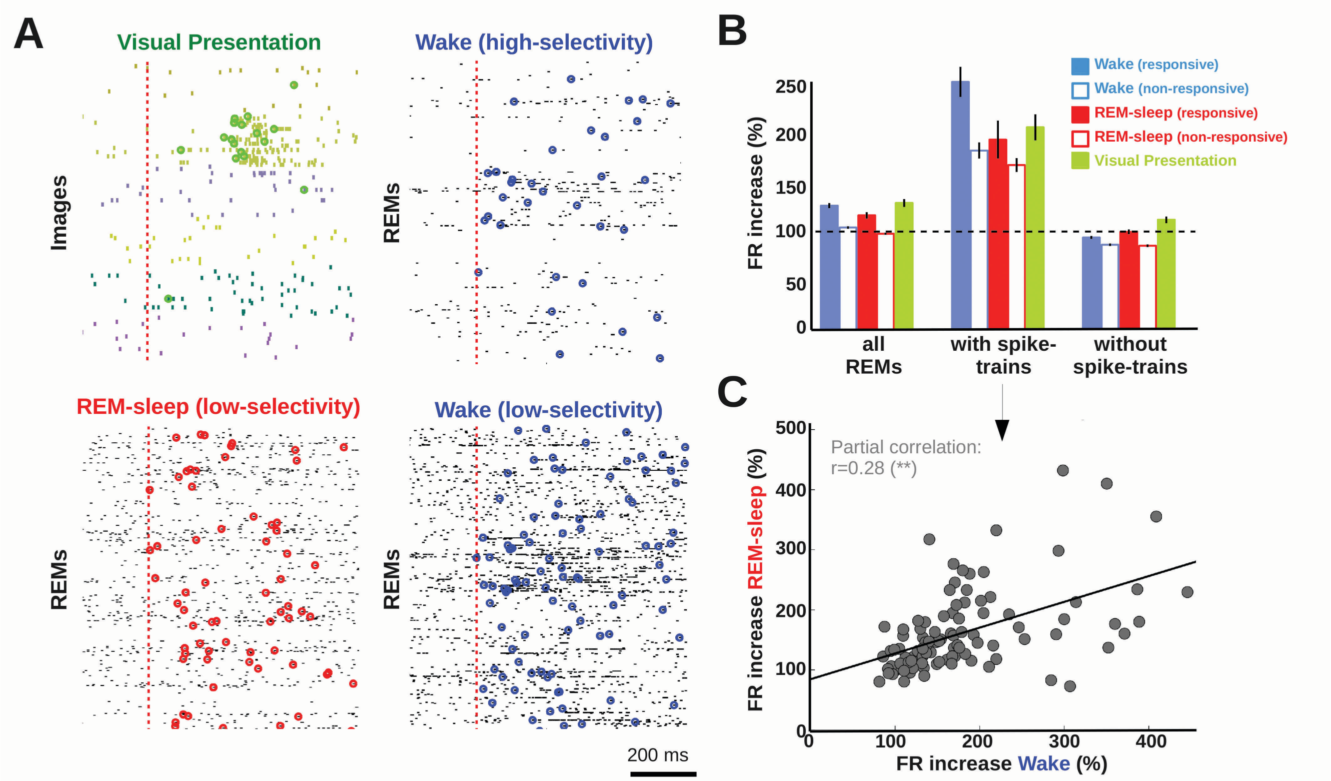

(A) Raster plots of representative single-unit responses following image presentation (top left) or REMs in wakefulness (top right, bottom right) and REMs during REM-sleep (bottom left). Dotted vertical red line denotes the time of image presentation or detected REM onset. Each spike is marked by a vertical tick. For the visual presentation, 6 different images were presented and the responses are grouped by image where the color of each spike corresponds to a different image. In other subpanels, trials are ordered chronologically from first (bottom) to last (top) REM. Circles show the onset of the detected spike-trains after time zero. Note the selectivity of the unit in the visual presentation paradigm (spike-trains detected almost exclusively for one image in green). Similar high ‘selectivity’ could be observed for REMs in wakefulness (top right). Other units exhibited low ‘selectivity’ during REMs, showing spike-trains for a larger percent of REMs in the few hundred milliseconds following REM onset (bottom). (B) Increase in firing rates for responsive MTL neurons (filled bars, N=50 in wakefulness, N=19 in REM-sleep) or non-responsive neurons (unfilled, N=132 units in wakefulness, N=113 units in REM-sleep) for all REMs (left), REMs with detected spike-trains (middle) or without (right). Green bars show the equivalent for MTL units in response to images (N=70). Note that REMs associated with spike-trains accounted well for the average post-REM increase in neuronal activity (as seen in Figure 2D). Error bars denote SEM across units. (C) Correlation of post-REM responses across vigilance states for those MTL neurons recorded in both wakefulness and REM-sleep (N=131 units) and for REMs with spike-trains. A partial correlation coefficient was estimated (Spearman's correlation) to establish the effect above and beyond differences in the mean firing rate of individual units (r=0.28, p<0.01 (**)). Thus, the magnitude of neuronal modulations was correlated across REM-sleep and wakefulness.

Supplementary Figure 4. Amplitude and duration of detected REMs

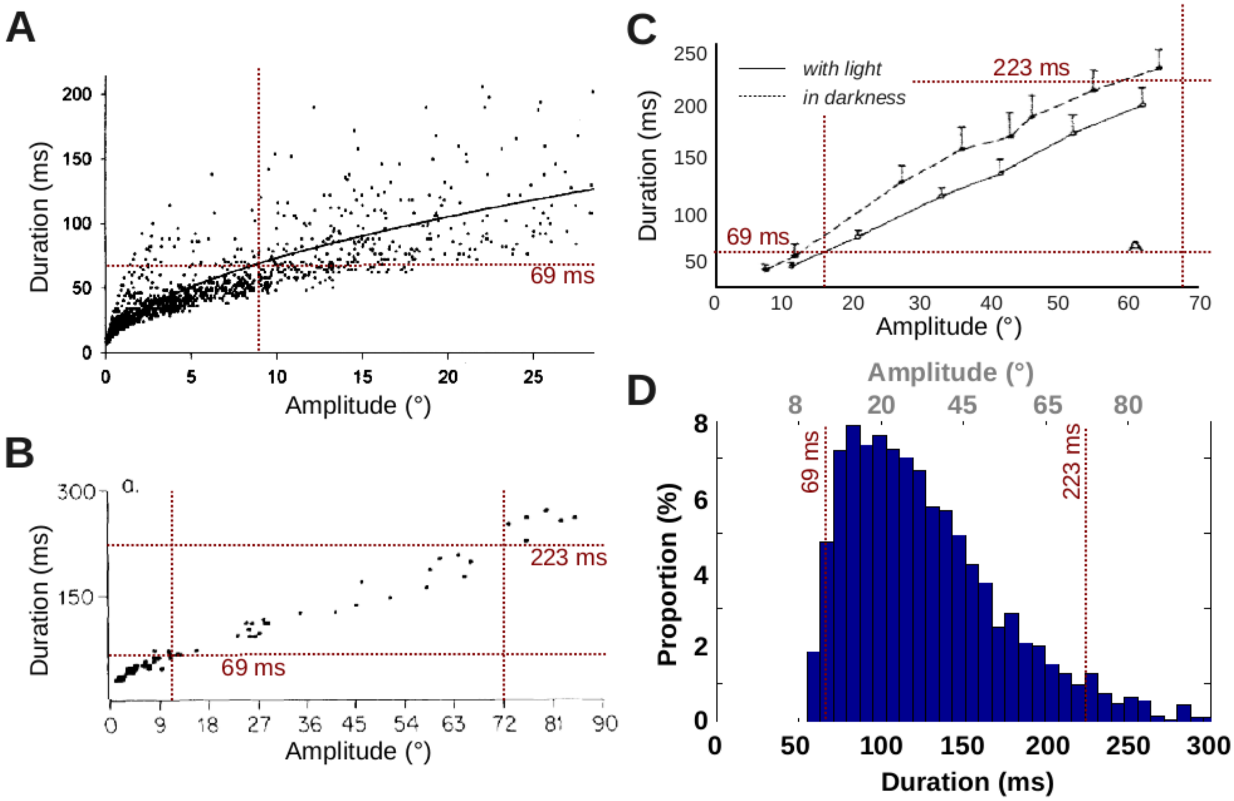

(A-C) For saccades larger than 5°, there is a linear relationship between saccades amplitude and duration (Panel A adapted from [12], Panel B from [11], Panel C from [10]). Red lines show the 5th and 95th percentile of REMs duration as detected in our dataset during wakefulness (n=2679 REMs). (D) Distribution of REMs duration: 90% of REMs lasted between 69 and 223ms. On top, an approximate scale gives the equivalence in deviation amplitude computed from panel A and B.

Supplementary Figure 5. Temporal dynamics of saccades and detected REMs

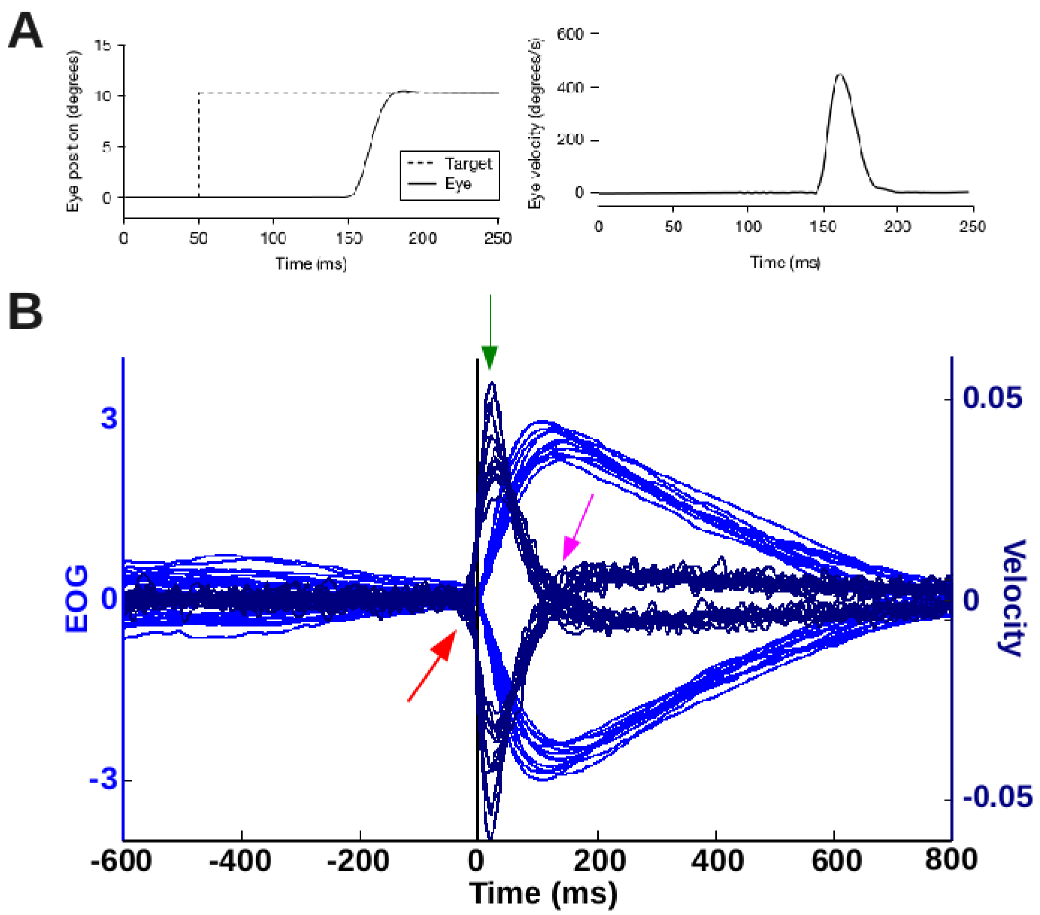

(A) Adapted from [5], schematic representation of the saccadic dynamics. At 150ms after target presentation, a 10° saccade is made, lasting for ~30ms. This corresponds to a sudden increase in velocity. Velocity goes back to 0 by the time the eye position is stabilized to the new target. (B) EOG amplitude and EOG 1st derivative triggered-average for REMs detected in wakefulness and averaged by subject (N=13, time-locked to REMs onset). The amplitude and 1st derivative were computed on the EOG signal z-scored on the [-600, 800]ms time-window for each REM. The red arrows show saccade onset and velocity initial increase. The green arrow shows the velocity peak. The pink arrows show the saccade offset and new fixation, which corresponds to a null velocity and peak in EOG amplitude.

Supplementary Figure 6. Effect of band-pass filtering on REMs average traces

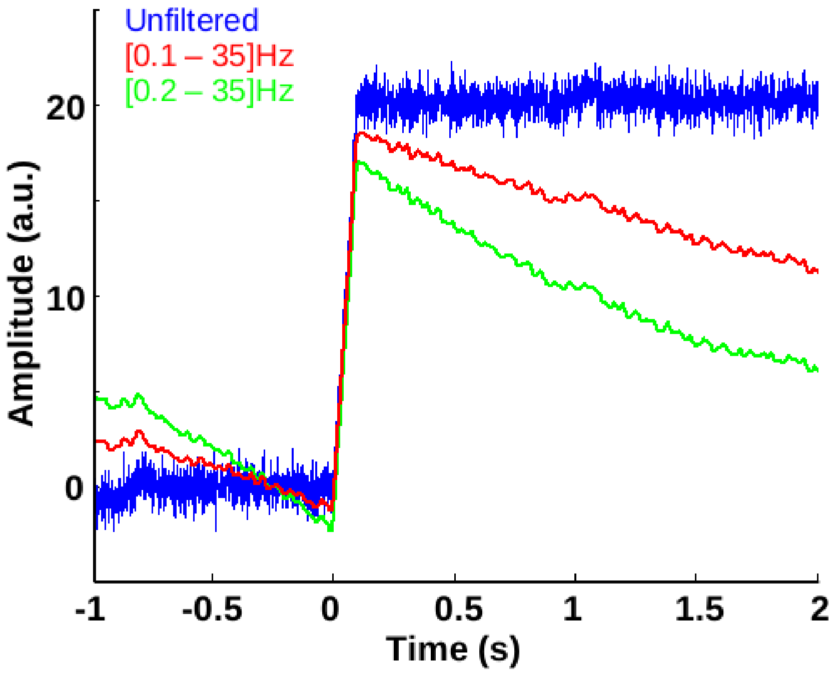

Triggered-average of simulated data (Gaussian noise with step function modeling EOG data with saccades) with or without band-pass filtering. Note that with band-pass filtering, the post-saccadic potential is not constant as in the unfiltered case (step function) but drifts toward 0.

Supplementary Note:
Relation between electro-oculography (EOG) and saccadic eye-movements

Recording saccades with EOG

Since its discovery, the EOG has been extensively used to monitor ocular movements and saccades in particular. EOG is a reliable non-invasive measurement of eye-movements and numerous studies established its relation with gold-standard techniques such as high-end video monitoring. Saccades are classically defined as “a rapid conjugate eye movement that shifts the line of sight (center of gaze) rapidly from one part of the visual field to another, mainly used for orienting towards an object of interest. It is characterized by stereotyped relationships between amplitude, duration, and peak velocity. In human subjects, peak velocity typically rises along with saccade amplitude up to a saturation level of 400–500° per second, which is reached when the amplitude exceeds 10–30°, whereas duration rises linearly at a rate of 1.5–3 ms per degree starting from a minimum of 20–30 ms”[1].

There are various methods to reliably record saccades made by an individual. The most accurate methods are the magnetic search coil[2] and video eye-tracking techniques[3-6]. However, these methods do not allow recording eye-movements with closed eyes or may cause major discomfort that render them unsuitable for prolonged sleep studies. To date, EOG is the most widely used technique to record saccadic eye movements with closed eyes[5,7]. EOG is a non-invasive indirect proxy that records movements of the eyes through the perturbation of electric currents generated by the eyes (corneo-retinal potentials)[3,5]. Importantly, many studies have already compared in detail EOG with direct measurement of eye-movements to assess the reliability and limitations of this technique.

With electrode placement (montage) and referencing as employed in our study, EOG reliably captures horizontal saccadic movements from 5° up to 70-80° of angle deviation[3,5,6,8,9]. In this approximate angle range, there exists a linear relationship between EOG and saccade amplitude[3,5,10–12]. However, the EOG is limited in the case of vertical eye-movements due to movements of the eye-lid[7,13,14], therefore our study focuses on horizontal eye-movements.

Thus, EOG captures horizontal saccades with medium angle deviations (that encompass the bulk of naturally occurring saccades[10]) and, within this range, the amplitude of the EOG signal can be used to infer about saccade amplitude. Ideally, the EOG is frequently calibrated against eye tracking so that the amplitude of each saccade can be estimated accurately from EOG signals (with a precision of about 0.5°[5]). Despite good inter-subject reliability[11], calibration requires the participant to periodically follow tasks with open eyes, and was therefore not feasible in the current sleep study. However, saccade duration and amplitude are tightly correlated with good inter-subject reliability[11,15]. Duration represents a more robust estimate since it does not depend on the absolute EOG voltage and can thus be used to indirectly assess saccades amplitude.

Given the linear relationship between EOG duration and saccade amplitude in degrees, we estimated the amplitude of detected REMs by computing the duration of each REM (defined as the time interval between REM onset and REM peak in the EOG signal). Supplementary Figure 4 shows the distribution of REM durations, and the estimated saccade amplitude based on the established linear relationship. As

can be seen, 90% of saccades were with durations within the 69-223ms range, corresponding to saccades between ~10° and ~70° of angle deviation. In line with the literature and the details of our algorithm, none of our detected REMs had durations shorter than 50ms (corresponding to saccades below 10° of angle deviation). All in all, the events detected in our study represent a broad range of saccades with angles mostly in the range of 10°-70°.

Detected REMs and their relation to saccades

In our study we recorded EOG signals using a standard montage that is recommended by the American Academy of Sleep Medicine (AASM) to record horizontal eye-movements during sleep studies[16], as well as for recordings of horizontal saccades[5]. We placed 2 electrodes on the right and left canthi referenced to earlobe electrodes, and the signals were band-pass filtered between 0.1 and 35Hz. Our own analysis supports the conclusion of previous studies that the specific filter settings do not significantly influence the detection outcome[9]. Using a semi-automatic algorithm, we detected rapid eye movements in wake and sleep from EOG recordings (see Figure 1D, Methods section and Supplementary Material). The shape of detected events (see single events in Figure 1G, average in Figure 1F) is in very good agreement with EOG traces as described in those studies that directly compared EOG signals to saccadic eye movements with eye tracking[9,17]. Therefore, the specific parameters used here to detect REM events are ideal to maximize the correspondence with horizontal saccadic eye-movements as detected with other techniques.

Saccades are typically considered to occur a few times every second during wakefulness, and this may seem at odds with our detected REMs - occurring 2.6 times per minute (0.04 per second) on average during wakefulness (Figure 1E). However, several factors can account for this apparent discrepancy:

(1) Our recordings took place during overnight sleep studies, therefore some epochs during wakefulness correspond to time intervals in which patients were awake but with closed eyes in bed, not gazing around. Indeed, when focusing on 10s epochs in which *any* REMs were detected, the rate of REMs was found to be 0.2 ± 0.01 REMs *per second*, representing a 5-fold increase compared to all wakefulness epochs. As a comparison, a recent study using infrared (IR) eye-tracking to record saccades during free-viewing conditions reported an average inter-saccade interval of 0.73sec[18] (vs. 2.0s ± 0.1 in our study).

(2) Even when our participants were in fact awake with open eyes, they were looking around in a static hospital room with no specific instructions to explore a visual scene that could remain constant at moments,

(3) Our conservative detection was optimized to avoid false-positives and therefore probably underestimates the occurrence of REMs (entails false-negatives)

(4) As explained in the previous section, vertical eye-movements as well as small (<5°) and micro-saccades (<2°) are not reliably captured with EOG recordings, therefore our detected events constitute a subset of all saccades as recorded with video tracking.

Temporal dynamics of detected REMs and relation to saccades' dynamics

In the saccade research field, it is often customary to focus on saccade velocity (rather than amplitude/duration) i.e. to extract the 1st derivative[5], whereas in the sleep polysomnography field it is

customary to treat the EOG signal "as-is"[19-21]. The advantage of velocity is to clearly isolate the moment during which eyes are moving (non-null velocity). To better compare the temporal dynamics of REM reported here vs. saccades as classically described, we superimposed the EOG traces along with their 1st derivatives (as customary in saccade research). As illustrated in Supplementary Figure 5 (and in Figure 2 in the main manuscript) a saccade starts with a sharp increase in EOG velocity (Supplementary Figure 5, red arrow). The velocity reaches a peak (green arrow, after ~25ms) and decreases back to zero (pink arrow, end of saccadic movement). Thus, in terms of the ***EOG*** (light blue traces), the peak in the signal corresponds to the end of the saccade and the new fixation[9,17]. EOG velocity highlights the transient nature of saccades (about 100ms) and verifies that in EOG traces, saccade duration can be approximated as the time between the onset and the peak in EOG amplitude (as defined in our Methods section).

It seems that the late slow component of the EOG trace returning to baseline (after the peak in amplitude, around 100-600ms) occurs after the new fixation and is not part of the saccadic main sequence. This slow drift could either reflect after-saccade physiological processes (such as up-shooting[5,12], correction for head-movements[9] or subsequent saccades) or, alternatively, could reflect in part our high-pass filter settings (0.1Hz, see Supplementary Figure 6).

Supplementary Methods

Subjects

Nineteen patients with pharmacologically intractable epilepsy (ages 19-52, 11 females) underwent monitoring with depth electrodes for seizure foci identification and potential surgical treament[22]. Patients provided written informed consent prior to participation in the research study, under the approval of the Medical Institutional Review Board at the University of California, Los Angeles, USA. Electrode location was based only on clinical criteria, and Itzhak Fried performed all surgery. For each subject, localization of the seizure onset zone was based on recordings during hospital monitoring, in combination with prior functional and anatomical neuroimaging[23]. Thirteen patients participated in a full overnight sleep study that also included epochs of wakefulness, nine patients participated in a visual object recognition paradigm (four of these individuals participated in both experiments), and one individual participated in a paradigm directly comparing activity around eye movements in darkness, with closed eyes, towards visual targets and during visual stimulation with fixation during wakefulness.

Data Acquisition

For each patient, 8 to 12 flexible polyurethane depth electrodes (1.25 mm diameter, see Figure 1B) were placed in some of the following regions: hippocampus, amygdala, entorhinal cortex, parahippocampal gyrus, temporal gyrus, fusiform gyrus, temporo-occipital junction; anterior, middle and posterior cingulate; supplementary motor area, inferior frontal gyrus, orbitofrontal cortex, and parietal cortex. Electrode location varied between patients based on their clinical profiles (see Figure 1C for an overview). Electrode positions were verified using post-implant computed tomography (CT) co-registered with pre-implant magnetic resonance (MR) imaging (Brain Navigator, Grass-Telefactor Corp., Philadelphia, PA). Scalp and intracranial depth EEG data were continuously recorded, sampled at 2 kHz, bandpass-filtered in hardware between 0.1Hz and 500Hz and re-referenced offline to the mean signal of the earlobes electrodes. Scalp EEG (C3, C4, Pz and Fz), EOG, EMG, video, and behavioral observations were collected and preprocessed according to established guidelines for sleep study ploysomnography[16] (see Figure 1A). The two EOG electrodes (used for REM detection) were pasted below the left and above the right canthi and referenced to the contralateral ear lobe.

In addition, each depth electrode terminated in a set of eight insulated 40-µm platinum-iridium microwires (impedances 200 to 500 kΩ, see Figure 1B)[22]. Microwire signals were simultaneously recorded continuously (Cheetah Recording System; Neuralynx, Tucson, AZ for 10 patients; Neuroport Recording System; Blackrock, Salt Lake City, UT for 9 patients), sampled at 28 kHz (10 patients) or 30 kHz (9 patients), band-pass filtered in hardware between 1Hz and 9kHz, and referenced locally to a ninth non-insulated microwire.

Unit identification and spike sorting

Units were identified using the 'wave_clus' software package[24] as follows: (i) extracellular microwire recordings were highpass filtered above 300Hz, (ii) a 5 SD threshold above the median noise level was computed, (iii) detected events were categorized into either real clusters or noise based on the reliability of action potential waveforms and by the presence of a refractory period for single units, as in [23] (see Supplementary Figure 1A). The enduring nature of unit recordings throughout hours of

continuous recordings (sleep studies: ~7h) was assessed by ensuring that action potential waveforms, and inter-spike-interval distributions were conserved in 1-hour intervals and that clusters remained separable throughout the recording session (see Supplementary Figure 1B). Overall, 600 units were identified for full-night sleep/wake recordings (355 putative single units, 245 multi-unit clusters) and 1,334 units for the visual stimulation sessions (541 putative single units, 916 multi-unit clusters).

Polysomnographic sleep studies

Sleep recordings were conducted at a minimal interval of 12 hours from identifiable seizures, 48-72 hr after surgery, and lasted for about 7 hours between 23:00 and 06:00. Sleep-wake stages (wakefulness, NREM sleep stages N1-N3 and REM sleep) were scored according to established guidelines[16]. Additional information about sleep properties, hypnograms and power spectra of scalp EEG were can be found in previous publications[23,25]. Wakefulness data relate here to periods preceding or following sleep as well as awakenings during sleep. On average, during these night recordings, patients had 4.5 (SD=2.2) continuous epochs of, at least, 1 minute of data scored as wakefulness (mean duration: 17.8 min, SD=15.2).

Behavioral notes during sleep studies were carefully examined to putatively distinguish between 'visual' and 'non-visual' epochs of wakefulness. Accordingly, in eight participants we were able to identify epochs which were either mostly 'non visual' (participant had open eyes while seated or lying in bed in a dark room; a total of 140 w-REMs in 304 minutes, 10% of REMs), or mostly 'visual' (participant actively watching TV/DVD or interacting with other people in a well-lit room; a total of 1250 w-REMs in 355 minutes). In order to compare neuronal firing rates around these 'visual' and 'non-visual' events (Figure 3A middle), we randomly selected 140 'visual' w-REMs (among 1,250) to yield an equal number of 'visual' and 'non-visual' REMs (287 REMs for the dEEG analysis, Figure 3A bottom). Firing rates ±500ms around REM onsets were compared to the baseline activity ([-800, -500]ms) across bi-phasic units and REMs (see Methods and below for a definition of bi-phasic units).

Visual stimulation studies

Eight patients participated in 22 sessions of a visual object recognition paradigm (2 sets of 6 pictures each). When possible, pictures were chosen based on their effectiveness in eliciting responses in the recorded neurons by means of a 'visual screening' experiment performed earlier that day[26]. Each session included two blocks, lasting 12 minutes each. During each block, 4 face and 2 place images were presented on a laptop computer for 200ms while subjects performed a face/place task. Each picture was presented 24 times in a pseudo-randomized order, with pseudo-random inter-stimulus intervals of 2-8s (uniform distribution).

Wakefulness eye movement paradigm

One individual participated in an eye-movement paradigm including four different experimental conditions examined in separate blocks: (a) saccades in darkness with open eyes, (b) saccades in darkness with closed eyes, (c) saccades to pictures presented at the periphery, (d) image presentation at the center of fixation. In the first two blocks (saccades in darkness), each trial started with a central fixation (white cross displayed on a portable computer screen superimposed on a dark background for [300 to 600]ms). A tone was then played (3,000 or 1,000 Hz, duration: 400ms), such that the low/high

pitch tones instructed the patient to perform a horizontal saccade towards the left/right sides of the screen. The participant was instructed to maintain peripheral fixation for [1400 to 1700]ms until the appearance of a double beep (2,000 Hz, duration: 300ms) after which participant had to fixate at the central cross again, in preparation for the next trial. The next trials started after a [1600 to 1900]ms period and the central fixation cross was not visible while gaze was directed at the periphery. In each block (see above) a total of 30 saccades were made to each direction. In the 3rd block (saccades to pictures), trials likewise began with central fixation for [300 to 600]ms, after which a beep was played (2,000 Hz, duration: 400ms) and an image appeared at the periphery either to the left or right side of the screen. The subject was instructed to make a saccade toward the picture and to maintain the fixation for [1400 to 1700]ms until it disappeared. Then, the patient had to make a saccade back to the center where the central fixation cross re-appeared. Ten pictures were displayed 6 times each, randomly in either the left or right visual field. The next trial started after a [1600 to 1900]ms period. Finally, in the 4th block (vision with fixation), the participant was instructed to maintain fixation on a central cross while pictures appeared at random intervals. The participant followed a face/place categorization task by pressing two keyboard buttons; ten images (5 places, 5 faces) were presented 6 times each.

Detection of Rapid-Eye Movements (REMs)

We used EOG recordings as a proxy to track saccadic eye movements in wakefulness and sleep. EOG constitutes a reliable indirect measure of eye position even through closed eye-lids (see Supplementary Note). REMs were detected in a semi-automatic manner as illustrated in Figure 1D. The two EOG signals (Figure 1D, 1st row, blue and red curves) were first band-pass filtered between 0.1 and 3 Hz using a zero-phase 2nd-order Butterworth filter, to attenuate fast background activity (Figure 1D, 2nd row). A detection threshold was then set at mean + 2 SD of the EOG signals across the whole night (epochs with absolute amplitude over 1,000μV were discarded from this computation so that such abnormal voltage would not influence our estimation) (Figure 1D, 2nd row). Events crossing this threshold were considered REMs candidates (Figure 1D, 3rd row) and further inspected so that (1) the segments above threshold had a duration of less than 1.5s; (2) they corresponded to an amplitude exceeding an equivalent threshold in the other EOG trace and of *opposite* sign; (3) their maximal slope (temporal 1st derivative) was above a threshold of 1μV/ms (Figure 1D, 4th row). We discarded REMs too close to each other (<0.5s) as well as REMs in close vicinity (within 0.5s) with detected epileptiform interictal spikes (rare). However, REMs tended to cluster in close succession, as illustrated in Figure 1G. Accordingly, 30±3% and 30±4% of REM onsets appeared within 1s from another REM onset in wakefulness and REM-sleep, respectively, Consequently, in both wakefulness and REM-sleep, the typical inter-REM interval was shorter than the expected value when considering uniform REM density across time (around 2.0s on average in wakefulness, see Supplementary Note and Figure 1). Possible residues of epileptiform interictal spikes were identified in the EOG signal by searching for abnormal increase (absolute amplitude superior to mean + 10 SD) in the 100-150 Hz band (Figure 1D, 5th row). We converged on these parameters after careful visual examination of the detected REMs and rejected candidates. We selected only the EOGs deflections with opposite phases since they correspond, in our EOG montage, to horizontal eye movements. Signal deflections that were in-phase across the two EOG traces were not analyzed since these may reflect either vertical eye movements or cortical potentials such as slow waves “leaking” to EOG measurements. REM onsets were defined automatically as the first crossing of the 2 raw EOGs traces before detected REMs (Figure 1D, 6th row). To ensure the quality of this detection scheme, *all* the automatically detected REMs and respective onset times were visually inspected. REM onsets were used as reference time points throughout our

analysis, but considering other time (peaks of REMs, times of maximal slope) did not significantly alter the main results reported. In order to minimize the influence of outliers on inter-subjects or inter-units averaging, epochs of REM sleep and wakefulness with less than 20 REMs were discarded for each patient from subsequent analysis (except for the 'visual' vs. 'non-visual' comparison). In addition to this detection, random epochs were selected to be used as a control data set. For each identified REM, we detected nearby 2s epochs (within 10s of real REMs) during which there was no significant increase in either EOG channel and no traces of epileptiform interictal spikes. These "random" events matched REMs detection in terms of frequency of occurrence, sleep stages and presence within the ultradian cycle (i.e. time within sleep) for each patient.

Evoked potentials in scalp and depth EEG

Evoked potentials for depth EEG channels (dERP) in the MTL were computed as follows. Depth EEG channels were segmented in 10s segments, notch-filtered (2nd-order IIR notch filter at 60 Hz and harmonics) to eliminate electrical noise, bandpass-filtered (4th-order Butterworth filter between 0.1 and 30Hz) and corrected for baseline activity by subtracting the mean amplitude across 10s segments. Epochs around REMs onsets were selected ([-800 1000] ms) and averaged by channel and vigilance state. Statistical deviance between dERPs (around REMs) and baseline ([-800, -400]ms) was assessed using a paired t-test (alpha=0.05, n=11 patients) on fixed windows (pre-REMs: [-50 0]ms, post-REMs: [50 200]ms and late-component: [300 400]ms in the sleep study, [200 600]ms for image presentation) in order to match common components across conditions.

Analysis of spiking activities during REMs

A triggered-averaging analysis computing peri-REM-time histograms (PRTH) was performed for each unit separately as follows. Spike data were time-locked to REM onsets and averaged across REMs (Figure 2D,E) or units (Supplementary Figure 2) on a [-800, 1000]ms window with 100ms bins (50ms overlap). Only REMs showing at least one spike in the [-800, 1000]ms window were included in PRTHs (for MTL units: n=16,738 events in wakefulness and 4,510 in REM-sleep). Individual neuronal activity was normalized on a [-800, -400]ms window and expressed as a percentage of baseline activity. In Supplementary Figure 2, MTL and non-MTL units were averaged together. Only units with at least 10 remaining REMs were included in the PRTH for Supplementary Figure 2.

When considering all units from all recorded brain regions (Supplementary Figure 2, top), a clear bi-phasic pattern appeared in wakefulness with a reduction in neuronal activity just before REM onset akin to saccadic suppression evidenced in monkeys[27] ([-100, -50]ms) followed by an increase in neural activity. This increase lasted for about 500ms after REM onsets, which is in accordance with latencies observed in MTL units in response to visual stimuli[28]. Interestingly, a similar (albeit smaller) increase was observed in REM-sleep between unit discharges after REM onsets ([50, 400]ms) and either (i) pre-onset activity ([-100, -50]ms): one-tailed t-test $p<0.01$) or (ii) baseline ($p<0.05$). Significant modulations were then detected unit-by-unit by comparing each bin separately to PRTHs of the same unit computed around random epochs via bootstrapping: for each time-interval, the real firing rate around REMs was compared to surrogate values computed around random epochs across 500 iterations and compared via a paired Mann-Whitney U-test ($\alpha=0.005$, uncorrected). To facilitate averaging between low- and high-firing rate neurons, the mean PRTH across all modulated neurons was computed after normalization of each neuron's PRTH by the average firing rate over the entire window

([-800, 1000ms]). Supplementary Figure 2 (middle row) shows the emergence of a bi-phasic modulation pattern around REMs (activity over [50, 400]ms > [-100, 50]ms or baseline activity, one-tailed t-tests, $p<0.05$) despite the fact that our selection criterion did not impose a particular directionality.

Subsequently, in order to focus on units showing a significant bi-phasic modulations resembling the pattern emerging from the neuronal population, we specifically selected units showing a significant increase in neuronal activity by comparing the average firing rate across REMs per unit and per sleep stage before ([-500, 0]ms) and after ([100, 600]ms) REM onsets (one-tailed sign-test, $\alpha=0.1$). Supplementary Figure 2 (bottom row) shows the equivalent PRTHs for all units from all recorded brain regions and Table 1 summarizes the proportion of modulated and bi-phasic units in the MTL and frontal regions (496 out of 600 units, the remaining units recorded in other temporal/parietal regions). Since bi-phasic units were more observed within the MTL, we restricted our subsequent analysis (Figures 2-5 in main text) to these "bi-phasic" units recorded in MTL regions. Figure 2D shows the PRTHs for all bi-phasic MTL units. To ensure the robustness of the bi-phasic pattern obtained in Figure 2D, statistical tests across bins were controlled for multiple comparisons using a cluster-permutation approach[29]. Figure 2D show the presence of significant positive clusters after REMs onset in wakefulness and REM-sleep (Monte-Carlo p-value<0.0001, cluster threshold: $p<0.05$) preceded by a decrease in firing rate prior to REMs onset (Monte-Carlo p-value<0.0001 in wakefulness, $p<0.05$ in REM sleep, cluster threshold: $p<0.1$). In the visual stimulation task, we observed only a positive cluster after image onset (Monte-Carlo p-value<0.0001, cluster threshold: $p<0.05$).

Analysis of spike-train latency and selectivity

Existence and latencies of spike-trains in individual trials (picture presentations or eye movements) were determined by Poisson spike train analysis as in 28. In this procedure, the inter-spike intervals (ISIs) of a given unit are processed continuously over a [-800, 1000]ms window and the onset of a spike-train is detected based on its deviation from a baseline Poisson, i.e., exponential, distribution of ISIs. Only units with mean firing rate above 2 Hz were included in this analysis (i.e. units behaving as a Poisson-process). For each trial (either picture presentation in controlled visual experiment or REM in wakefulness or REM in sleep) we determined the presence of spike-trains in this trial and the latency as the time between REM/image onset and the onset of the first spike in the spike-train. Only latencies within the first 1000ms were considered and the overall latency of that neuron was taken as the mean across all trials. Selectivity was defined as 100% minus the percentage of trials (either REMs or visual stimuli) for which a spike-train was detected. In the visual presentation paradigm, this value was restricted to limited set of fixed values due to the fact that a set of 6 images were presented to each patient. In Figure 4 and Supplementary Figure 3, all units within the MTL were analyzed and not only bi-phasic units. Comparison between individual neurons in wake and sleep focused on those neurons that were recorded in both vigilance states (n=131, Figure 4D).

Analysis of phase consistency of intracranial EEG

Phase consistency across REMs was estimated as follows. First, we performed a time-frequency decomposition of the intracranial dEEG signal (EEGlab toolbox[30]) using a standard Fast-Fourier Transform on a 384ms window and a padding ratio of 2. Then the phase of the signal was extracted for

all REMs/images and for each time and frequency. Phase coherence across REMs/image presentation was then computed using Euler's formula by time and frequency:

$$ITCP_{t,f}=\sqrt{\left(\frac{1}{n}\left(\sum^{n}\cos(\varphi_{t,f})\right)^{2}+\frac{1}{n}\left(\sum^{n}\sin(\varphi_{t,f})\right)^{2}\right)}$$

(ITCP: inter-trial phase coherence; n: number of REMs/images; $\varphi_{t,f}$: phase of the dEEG signal at time t and frequency f)

Due to the circularity of phase, a uniform distribution of phases across trials leads to a null phase coherence value. However, the repeated occurrence of a particular phase across trials (REMs or images), regardless of its exact value, leads to a non-null phase coherence value (with a maximum at 1 when the exact same phase recurs from trial to trial). Therefore, this procedure provides a measure of the re-occurrence across all trials of a specific phase at the same time relative to trial onset. Each time and frequency point was then compared to the phase coherence within a [-507, -151]ms baseline window (paired t-test, False-Discovery Rate (FDR) correction for multiple comparison) in wakefulness (N=71 dEEG channels), REM sleep (N=59) and image presentation (N=102, see Figure 5). Correlation across vigilances states was assessed by means of Pearson's correlation method by averaging phase coherence over a [2, 6]Hz window and [0, 400]ms window after REMs onset in wake and sleep. Given that ITCP depends on the number of events used to estimate it (see equation above), the number of REMs were equated across wake and sleep for each dEEG channel (N=50 REMs per channel, N=57 channels).

Statistics

Error bars in all figures denote standard error of the mean (SEM = SD/√(n-1), where n is the number of data points) unless otherwise stated. Student T-tests were performed after confirming normal distributions via Kolmogorov-Smirnov tests. Mann-Whitney U-tests (non-parametric) were used when the normality was not confirmed. Correlations were examined using the non-parametric Spearman's rank correlation coefficient or Pearson's method for normally distributed pairs of variables. When necessary, corrections for multiple comparisons were performed using the False Discovery Rate (FDR) method[31] or using a non-parametric cluster-permutation approach[29].

Supplementary References

1. Squire, L. R. *Encyclopedia of neuroscience*. (Academic Elsevier, 2009).
2. Robinson, D. A. A method of measuring eye movement using a scleral search coil in a magnetic field. *IEEE Trans Biomed Eng* **10**, 137–145 (1963).
3. Young, L. R. & Sheena, D. Survey of eye movement recording methods. *Behavior Research Methods & Instrumentation* **7**, 397–429 (1975).
4. Carpenter, R. H. S. *Movements of the eyes*. (Pion, 1988).
5. *Neuro-ophthalmology: neuronal control of eye moments*. (Karger, 2007).
6. Marg, E. Development of electro-oculography; standing potential of the eye in registration of eye movement. *AMA Arch Ophthalmol* **45**, 169–185 (1951).
7. Iwasaki, M. *et al.* Effects of eyelid closure, blinks, and eye movements on the electroencephalogram. *Clin Neurophysiol* **116**, 878–885 (2005).
8. Joyce, C. A., Gorodnitsky, I. F., King, J. W. & Kutas, M. Tracking eye fixations with electroocular and electroencephalographic recordings. *Psychophysiology* **39**, 607–618 (2002).
9. Boulanger, M., Galiana, H. L. & Guitton, D. Human eye-head gaze shifts preserve their accuracy and spatiotemporal trajectory profiles despite long-duration torque perturbations that assist or oppose head motion. *Journal of Neurophysiology* **108**, 39–56 (2012).
10. Becker, W. & Fuchs, A. F. Further properties of the human saccadic system: eye movements and correction saccades with and without visual fixation points. *Vision Res.* **9**, 1247–1258 (1969).
11. Baloh, R. W., Sills, A. W., Kumley, W. E. & Honrubia, V. Quantitative measurement of saccade amplitude, duration, and velocity. *Neurology* **25**, 1065–1070 (1975).
12. Leigh, R. J. Using saccades as a research tool in the clinical neurosciences. *Brain* **127**, 460–477 (2003).
13. Ford, A., White, C. T. & Lichtenstein, M. Analysis of eye movements during free search. *J Opt Soc Am* **49**, 287–292 (1959).
14. Yee, R. D. *et al.* Velocities of vertical saccades with different eye movement recording methods. *Invest. Ophthalmol. Vis. Sci.* **26**, 938–944 (1985).
15. Bahill, A. T., Clark, M. R. & Stark, L. The main sequence, a tool for studying human eye movements. *Mathematical Biosciences* **24**, 191–204 (1975).
16. Iber, C., Ancoli-Israel, S., Chesson, A. & Quan, S. The AASM Manual for the Scoring of Sleep and Associated Events: Rules, Terminology and Technical Specifications. (2007).
17. Plöchl, M., Ossandón, J. P. & König, P. Combining EEG and eye tracking: identification, characterization, and correction of eye movement artifacts in electroencephalographic data. *Frontiers in Human Neuroscience* **6**, (2012).
18. Wang, H. X., Freeman, J., Merriam, E. P., Hasson, U. & Heeger, D. J. Temporal eye movement strategies during naturalistic viewing. *Journal of Vision* **12**, 16–16 (2012).
19. Herman, J. H., Barker, D. R. & Roffwarg, H. P. Similarity of eye movement characteristics in REM sleep and the awake state. *Psychophysiology* **20**, 537–543 (1983).
20. Doricchi, F., Iaria, G., Silvetti, M., Figliozzi, F. & Siegler, I. The 'ways' we look at dreams: evidence from unilateral spatial neglect (with an evolutionary account of dream bizarreness). *Exp Brain Res* **178**, 450–461 (2007).
21. Jacobs, L., Feldman, M. & Bender, M. B. Are the eye movements of dreaming sleep related to the visual images of the dreams? *Psychophysiology* **9**, 393–401 (1972).
22. Fried, I. *et al.* Cerebral microdialysis combined with single-neuron and electroencephalographic recording in neurosurgical patients. Technical note. *J Neurosurg* **91**, 697–705 (1999).
23. Nir, Y. *et al.* Regional slow waves and spindles in human sleep. *Neuron* **70**, 153–69 (2011).
24. Quiroga, R. Q., Nadasdy, Z. & Ben-Shaul, Y. Unsupervised spike detection and sorting with wavelets and superparamagnetic clustering. *Neural Comput* **16**, 1661–87 (2004).
25. Andrillon, T. *et al.* Sleep Spindles in Humans: Insights from Intracranial EEG and Unit Recordings. *J Neurosci* **31**, 17821–17834 (2011).

26. Quiroga, R. Q., Reddy, L., Kreiman, G., Koch, C. & Fried, I. Invariant visual representation by single neurons in the human brain. *Nature* **435**, 1102–1107 (2005).
27. Bremmer, F., Kubischik, M., Hoffmann, K.-P. & Krekelberg, B. Neural Dynamics of Saccadic Suppression. *Journal of Neuroscience* **29**, 12374–12383 (2009).
28. Mormann, F. et al. Latency and selectivity of single neurons indicate hierarchical processing in the human medial temporal lobe. *Journal of neuroscience* **28**, 8865-8872 (2008).
29. Maris, E. & Oostenveld, R. Nonparametric statistical testing of EEG- and MEG-data. *J. Neurosci. Methods* **164**, 177–190 (2007).30. Delorme, A. & Makeig, S. EEGLAB: an open source toolbox for analysis of single-trial EEG dynamics including independent component analysis. *J. Neurosci. Methods* **134**, 9–21 (2004).
31. Benjamini, Y. & Yekutieli, D. The control of the false discovery rate in multiple testing under dependency. *Ann. Statist.* **29**, 1165–1188 (2011).
